# Cardioprotective effects of angiotensin III against ischemic injury via the AT_2_ receptor and K_ATP_ channels

**DOI:** 10.1002/phy2.151

**Published:** 2013-11-13

**Authors:** Byung Mun Park, Shan Gao, Seung Ah Cha, Byung Hyun Park, Suhn Hee Kim

**Affiliations:** 1 Department of Physiology, Research Institute for Endocrine Sciences, Chonbuk National University Medical SchoolJeonju, Korea; 2 Department of Biochemistry, Research Institute for Endocrine Sciences, Chonbuk National University Medical SchoolJeonju, Korea

**Keywords:** Angiotensin III, apoptosis, atrial natriuretic peptide, Bax, Bcl-2, caspase-3, caspase-9, catalase, heart, heme oxygenase-1, ischemia, K_ATP_ channel, superoxide dismutase

## Abstract

Angiotensin III (Ang III) has similar effects on blood pressure and aldosterone secretion as Ang II, but cardioprotective effects are also proposed. In this study, we investigated whether Ang III protects the heart against ischemia/reperfusion (I/R) injury. After sacrificing Sprague-Dawley rats, the hearts were perfused with Krebs–Henseleit buffer for a 20 min preischemic period with and without Ang III followed by 20-min global ischemia and 50-min reperfusion. Pretreatment with Ang III (1 *μ*mol/L) improved an increased postischemic left ventricular end-diastolic pressure (LVEDP) and a decreased postischemic left ventricular developed pressure (LVDP) induced by reperfusion compared to untreated hearts. Ang III markedly decreased infarct size and lactate dehydrogenase levels in effluent during reperfusion. Ang III increased coronary flow and the concentrations of atrial natriuretic peptide in coronary effluent during reperfusion. Pretreatment with Ang II type 2 receptor (AT_2_R) antagonist or ATP-sensitive K^+^ channel (K_ATP_) blocker for 15 min before ischemia attenuated the improvement of LVEDP, LVDP, and ±d*P*/d*t* induced by Ang III. Ang III treatment increased Mn-superoxide dismutase, catalase, and heme oxygenase-1 protein levels, which was attenuated by pretreatment with AT_2_R antagonist or K_ATP_ blocker. Ang III treatment also decreased Bax, caspase-3, and caspase-9 protein levels, and increased Bcl-2 protein level, which were attenuated by pretreatment with AT_2_R antagonist or K_ATP_ blocker. These results suggest that the cardioprotective effects of Ang III against I/R injury may be partly related to activating antioxidant and antiapoptotic enzymes via AT_2_R and K_ATP_ channels.

## Introduction

The renin–angiotensin system (RAS) plays important roles in the regulation of blood pressure and renal function. Angiotensin (Ang) II is a major effective peptide of RAS. Ang II is degraded by aminopeptidase A and N to Angiotensin III (Ang III) and Ang IV, respectively (Reaux et al.^2001^; Carey and Siragy 2003). These metabolites are known to exert biological activities. Ang III has similar functions to Ang II, such as aldosterone secretion (Campbell et al. 1974; Yatabe et al. 2011), vasopressin release (Zini et al. 1996), DNA and collagen synthesis in cardiac fibroblast, and water intake (Wilson et al. 2005). However, Ang III has different or opposite functions to Ang II, such as sodium excretion (Padia et al. 2009).

Recently, we reported an inhibitory effect of Ang II on atrial natriuretic peptide (ANP) secretion via the Ang II type 1 receptor (AT_1_R) (Oh et al. 2011a,b) and a stimulatory effect of Ang III on ANP secretion via Ang II type 2 receptor (AT_2_R) in highly stretched atria and in vivo experiments (Park et al. 2013). ANP is a well-known cardiac peptide hormone with beneficial effects on cardiovascular and renal diseases. Based on results showing a stimulatory effect of Ang III-induced ANP secretion, we hypothesized that Ang III may have cardioprotective effects opposite to those of Ang II. Especially, an agonist of the mitochondrial ATP-sensitive K^+^ (K_ATP_) channel mimics ischemic preconditioning (Garlid et al. 1997), while its antagonist prevents diazoxide-mediated cardioprotection (Auchampach et al. 1992). Ang II acts at G(q/11)-coupled receptors to suppress K_ATP_ channel activity in rat arterial smooth muscle (Hayabuchi et al. 2001). However, it is not known whether Ang III has cardioprotective effects against ischemia/reperfusion (I/R) injury and these effects are related to opening of K_ATP_ channel activity. The aim of this study was to define the effects of Ang III on I/R injury and its signaling pathways using an isolated perfused Langendorff rat heart model.

## Material and Methods

### Experimental animals and drugs

Male Sprague-Dawley rats, weighing 200–250 g, were obtained from Daehanbiolink (Eumsung, Korea) and housed in a temperature-controlled room with a 12:12-h light-dark cycle. Animals were provided free access to standard laboratory chow (5L79 Purina rat and mouse 18% chow; Charles River Laboratories Inc., Wilmington, MA) and water. All of experimental protocols conformed to the National Institutes of Health Guide for the Care and Use of Laboratory Animals (NIH publication no. 85-23, revised 1996) and were approved by our institution.

Ang III was purchased from Bachem (Bubendorf, Switzerland) and was dissolved in distilled water. Loartan, PD123319 (PD), A779, and 5-hydroxydecanoic acid (5-HD) were purchased from Sigma-Aldrich (St. Louis, MO) and were dissolved in distilled water.

### Preparation of isolated perfused rat hearts

Rats were sacrificed by cervical dislocation. Each heart was immediately excised, and the ascending aorta was cannulated and immediately retrograde perfused on a Langendorff apparatus with Krebs–Henseleit (K–H) solution (in mmol/L: 119 NaCl, 4.7 KCl, 1.25 CaCl_2_, 1.24 MgSO_4_, 20.1 NaHCO_3_, 1.24 KH_2_PO_4_, and 11.2 glucose) at a constant pressure of 80 mmHg (Gao et al. 2012; Piao et al. 2010). The perfusate was bubbled with 95% O_2_ and 5% CO_2_, and then kept at 37°C. To assess contractile function, a latex balloon was connected to a pressure transducer laboratory (ML-820; ADInstruments Pvt. Ltd, Bella Vista, Australia) and inserted into the left ventricular cavity via the left atrium. Left ventricular end-diastolic pressure (LVEDP) was set at 5–10 mmHg by inflating the balloon with physiological saline and left ventricular pressure was then continuously recorded. Coronary flow was measured by collecting the effluent. Pacing wires were fixed to the right atrium and left ventricle, and hearts were paced at 5 Hz (2.0 V, 2.0 msec).

### Experimental protocols

**Figure 1:**
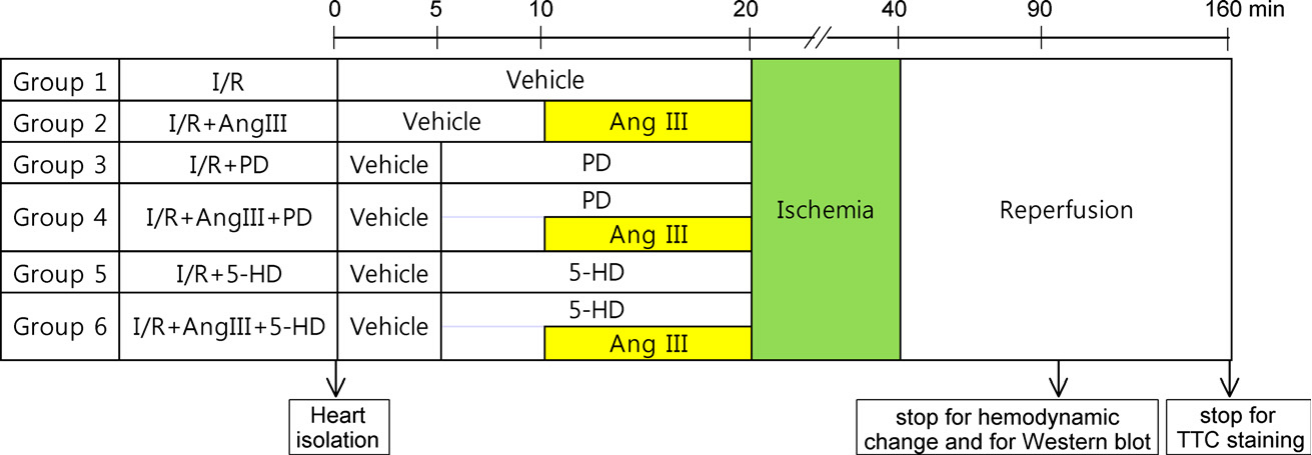
Experimental protocol to determine the effects of angiotensin III (Ang III) on ischemia–reperfusion injury. Ang III, Angiotensin III; PD, PD123319; 5-HD, 5-hydroxydecanoic acid; TTC, triphenyltetrazolium chloride.

Hearts were perfused for a total of 90 min (for the measurement of hemodynamic changes and western blot) or 160 min (for the measurement of myocardial infarct size) consisting of a 20-min preischemia period followed by a 20-min global ischemia and 50- or 120-min reperfusion at 37°C (Fig. 1). The following groups were studied. In group 1, hearts were perfused with K–H buffer for 20-min preischemia period followed by a 20-min global ischemia and 50-min reperfusion at 37°C (control group, I/R, *n* = 19). In group 2, hearts were perfused with K–H buffer for 20-min preischemia period in the presence of Ang III (1 *μ*mol/L) followed by a 20-min global ischemia and 50-min reperfusion at 37°C (I/R + Ang III, *n* = 15). In group 3, hearts were perfused with K–H buffer containing an AT_2_ receptor antagonist, PD123319, (1 *μ*mol/L), for 15-min preischemia period followed by a 20-min global ischemia and 50-min reperfusion at 37°C (I/R + PD, *n* = 6). In group 4, PD123319 was pretreated 5 min before Ang III application followed by a 20-min global ischemia and 50-min reperfusion at 37°C (I/R + Ang III + PD, *n* = 8). In group 5, hearts were perfused with K–H buffer containing a K_ATP_ channel blocker (5-HD) (10 *μ*mol/L) for 15-min preischemia period followed by a 20-min global ischemia and 50-min reperfusion at 37°C (I/R + 5-HD, *n* = 6). In group 6, 5-HD was pretreated 5 min before Ang III application followed by a 20-min global ischemia and 50-min reperfusion at 37°C (I/R + Ang III + 5-HD, *n* = 9) (Fig. 1). After finishing the experiments, the hearts were quick frozen in liquid nitrogen and then stored in −70°C for western blotting.

### Measurement of lactate dehydrogenase concentration in effluent

The severity of myocardial injury was determined by concentrations of lactate dehydrogenase (LDH) in the effluent. Effluents were collected every 5 min during the preischemic period and the first 40-min reperfusions from all groups and concentrations of LDH was assayed using a LDH ELISA kit (Takara Bio Inc., Otsu, Japan).

### Measurement of ANP concentration in effluent

Effluents were collected every 5 min during the preischemic period and the first 40-min reperfusion from the control and Ang III-treated groups. The ANP in effluent was extracted using Sep-Pak C_18_ cartridges (Cho et al. 1989), dried, and measured using a specific radioimmunoassay, as described previously (Cho et al. 1988).

### Determination of myocardial infarct size

After 120-min reperfusion, isolated hearts were removed from the Langendorff apparatus and frozen at −20°C for 1–2 h. The hearts were then sectioned (2–3 mm) and incubated in phosphate buffer (pH 7.4) that contained 0.75% triphenyltetrazolium chloride for 6 min at 37°C and fixed in 10% formalin. Infarcted areas were determined by planimetry (Analysis pro ver.3.2; Soft Imaging System GmH, Munster, Germany) and infarct size was calculated as the percentage of area-at-risk (% infarct size/area at risk [IS/AAR]) (Piao et al. 2010; Gao et al. 2012).

### Western blot analysis

Total proteins were extracted from the left ventricle of the heart. The samples were placed in lysis buffer (M-PER; Thermo, Rockford, IL) containing protease inhibitor, homogenized, incubated on ice for 30 min, and then centrifuged at 16,000*g* for 15 min. After determining protein concentrations in supernatant using a modified Bradford assay, 30 *μ*g of total protein was boiled in loading buffer for 5 min and loaded onto gradient sodium dodecyl sulfate polyacrylamide gels (SDS-PAGE). Following electrophoresis, proteins were transferred to an immobilon-polyvinylidene fluoride membrane. Membranes were blocked with Tris buffered saline-Tween 20 (TBS-T) skim milk powder for 1 h at room temperature. The membrane was incubated with primary antibodies against Bax, Bcl-2, caspase-3, caspase-9 (Cell Signaling Technology, Denver, MA), catalase (Calbiochem AG, San Diego, CA), Mn-superoxide dismutase (Mn-SOD), and heme oxygenase-1 (HO-1; Enzo Life Science, Plymouth Meeting, PA). Proteins were detected with horseradish peroxidase-conjugated secondary antibody for 1 h at room temperature. Immune reactivity was detected by chemiluminescence (Piao et al. 2010; Gao et al. 2012).

### Statistical analysis

All values are expressed as means ± SEM. Differences in hemodynamic variables between the control and treatment groups were assessed using analysis of variance followed by the Bonferroni multiple comparison test (GraphPAD Software, San Diego, CA). The significance level was *P* < 0.05.

## Results

### Effects of Ang III on ventricular hemodynamics during I/R injury

**Figure 2:**
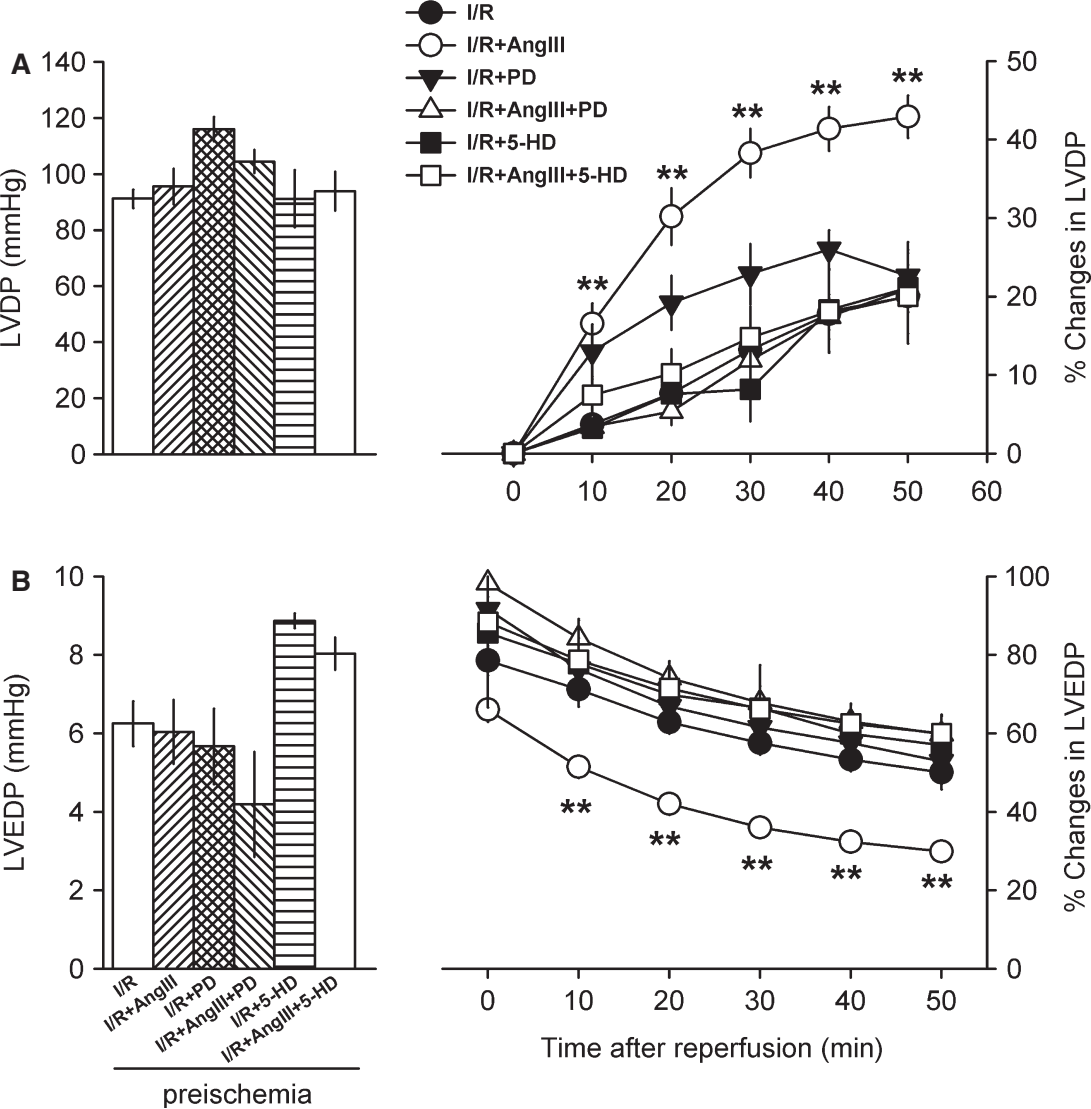
Time course of changes in left ventricular developed pressure (LVDP, A) and left ventricular end-diastolic pressure (LVEDP, B) by postischemia in control and Ang III-treated rat hearts. For each condition, basal preischemic values are shown as absolute values (left ordinate), and as% of the corresponding values taken over the preischemic value (right ordinate). I/R, ischemia and reperfusion only; I/R + Ang III, ischemia and reperfusion in Ang III-treated group; I/R + PD, ischemia and reperfusion in pretreatment with PD123319 (PD, 1 *μ*mol/L) for 15 min before ischemia in control group; I/R + Ang III + PD, ischemia and reperfusion in pretreatment with PD for 15 min before ischemia in Ang III-treated group; I/R + 5-HD, ischemia and reperfusion in pretreatment with 5-hydroxydecanoic acid (5-HD; 10 *μ*mol/L) for 15 min before ischemia in control group; I/R + Ang III + 5-HD, ischemia and reperfusion in pretreatment with 5-HD for 15 min before ischemia in Ang III-treated group. Values are mean ± SEM of 6–19 rats. **P < 0.01 vs. I/R group. I/R, ischemia/reperfusion; Ang III, Angiotensin III; PD, PD123319; 5-HD, 5-hydroxydecanoic acid.

**Figure 3:**
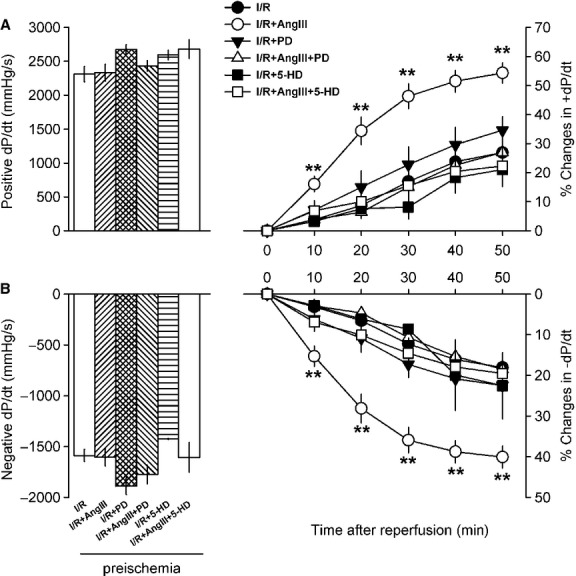
Time course of changes in +dP/d*t* (A) and –dP/d*t* (B) by postischemia in control and Ang III-treated rat hearts. Values are mean ± SEM of 6–19 rats. **P < 0.01 vs. I/R group. Ang III, Angiotensin III; I/R, ischemia/reperfusion.

Before the ischemic period, all measured parameters, such as left ventricular developed pressure (LVDP), LVEDP, ±dP/dt, and coronary flow, were comparable among groups. There are some individual variations in basal value. Therefore, we always did both control and experimental groups together. The time courses of changes in LVDP, LVEDP, ±dP/dt after 20-min global ischemia are shown in the absence or presence of Ang III (1 *μ*mol/L), PD, and 5-HD (Figs. 2 and 3). Previously, we reported that Ang III from 0.3 to 10 *μ*mol/L stimulates ANP secretion in a dose-dependent manner (Park et al. 2013). Therefore, we chose 1 *μ*mol/L Ang III even though the dose is relatively high. Values are shown as relative percent changes calculated using preischemic control values and experimental values during reperfusion. Postischemic LVDP appeared to recover slowly and reached 20.03 ± 2.00% of control value 50 min after the start of reperfusion (Fig. 2A). Postischemic LVDP in preexposure to Ang III was 42.90 ± 2.69% of the control value 50 min after the start of reperfusion. Pretreatment with Ang III improved the postischemic decrease in LVDP significantly compared to untreated control hearts at 10, 20, 30, 40, and 50 min of reperfusion (Fig. 2A). LVEDP abruptly increased after reperfusion by 80% of control value and then slowly decreased up to 50% of control value at 50 min. Pretreatment with Ang III improved postischemic increase in LVEDP significantly compared to untreated control hearts at 10, 20, 30, 40, and 50 min of reperfusion (29.90 ± 2.57% vs. 50.05 ± 3.07% of control value on 50 min) (Fig. 2B). The cardioprotective effects of Ang III against I/R injury were abolished in the presence of PD123319 or 5-HD (Fig. 2A and B).

Positive and negative dP/dt abruptly decreased after reperfusion and then slowly recovered to 26.91 ± 3.31% and −18.06 ± 1.99% of the control values at 50 min, respectively. Ang III treatment improved postischemic changes in ±dP/dt as compared to untreated control hearts at 10, 20, 30, and 40 min of reperfusion (Fig. 3A and B). At 50 min after reperfusion, Ang III treatment significantly improved postischemic ±dP/dt to 55.9 ± 3.47% and −39.6 ± 2.98% of the control value, respectively. Pretreatment with PD123319 or 5-HD for 15 min abolished improvement of postischemic ±dP/dt by Ang III (Fig. 3A and B). PD123319 or 5-HD alone did not result in any changes in cardiac functions in the control group.

### Changes in coronary flow, LDH activity, and ANP concentration in effluents

**Figure 4:**
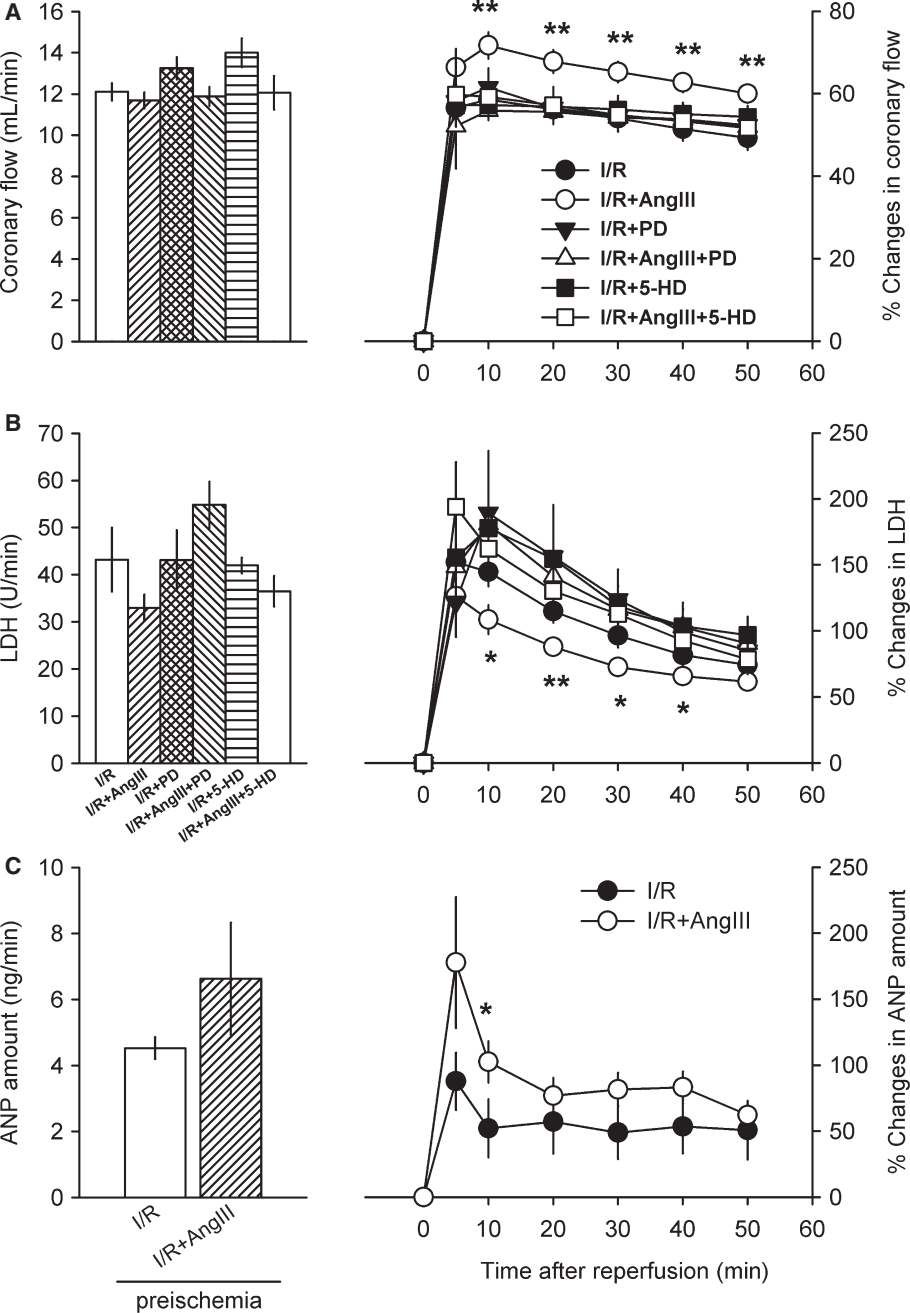
Time course of changes in coronary flow (A, *n* = 6–19), LDH activity (B, *n* = 5–7), and ANP concentration (C, *n* = 5) in coronary effluent by postischemia in rat hearts treated with Ang III. Values are mean ± SEM. *P < 0.05 vs. I/R group. LDH, lactate dehydrogenase; ANP, atrial natriuretic peptide; Ang III, Angiotensin III; I/R, ischemia/reperfusion.

Postischemic reperfusion decreased coronary flow by 50% of the control value and maintained constantly throughout experiments (12.11 ± 0.43 vs. 6.05 ± 0.49 mL/min on 50 min). Postischemic coronary flow was also improved by Ang III treatment and this effect was attenuated by pretreatment with PD123319 or 5-HD for 15 min (Fig. 4A). LDH activity in coronary effluent was abruptly increased at 5, 10, and 20 min after reperfusion. LDH levels during the postischemic period in the Ang III-treated group were significantly lower than in the control group, and this effect was attenuated by pretreatment with PD123319 or 5-HD for 15 min (Fig. 4B). PD123319 or 5-HD alone did not result in any changes in coronary flow and LDH level. Figure 4C shows changes in total amounts of ANP in coronary effluent before and after ischemia with and without Ang III. In control hearts, the total amount of ANP was relatively constant before and after ischemia. The total amount of ANP was higher at 10 min after reperfusion in the Ang III-treated group than in the control group (Fig. 4C).

### Changes in infarct size

**Figure 5:**
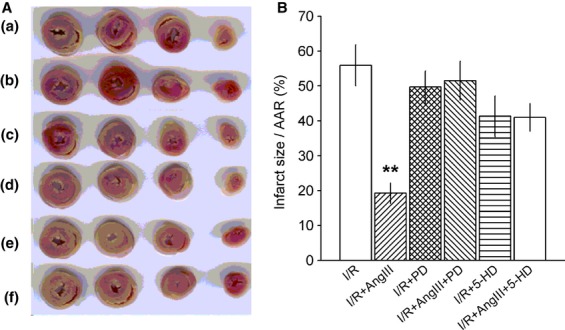
(A) Changes in ischemia/reperfusion-induced infarct size in vehicle (a), Ang III- (b), PD- (c), PD + Ang III- (d), 5-HD- (e), and 5-HD + Ang III- (f) treated hearts. (B) Infarct size was expressed as percent of the area-at-risk (AAR). Values are mean ± SEM of four rats of each group. **P < 0.01 vs. I/R group. Ang III, Angiotensin III; PD, PD123319; 5-HD, 5-hydroxydecanoic acid; I/R, ischemia/reperfusion.

To evaluate whether improvement of postischemic cardiac function by Ang III is related to morphological findings, the infarcted areas were measured 120 min after reperfusion. Infarct sizes in the control group were more extensive compared to the Ang III-treated group (Fig. 5A). Quantitative analyses of infract size showed that Ang III significantly decreased the sizes of infarcts induced by reperfusion injuries, from 55.89 ± 5.89% to 19.25 ± 2.93% IS/AAR, and that pretreatment with PD123319 or 5-HD completely attenuated this effect (Fig. 5B). PD123319 or 5-HD alone did not result in any significant changes in infarct size induced by I/R.

### Changes in antioxidant and apoptotic protein expressions

**Figure 6:**
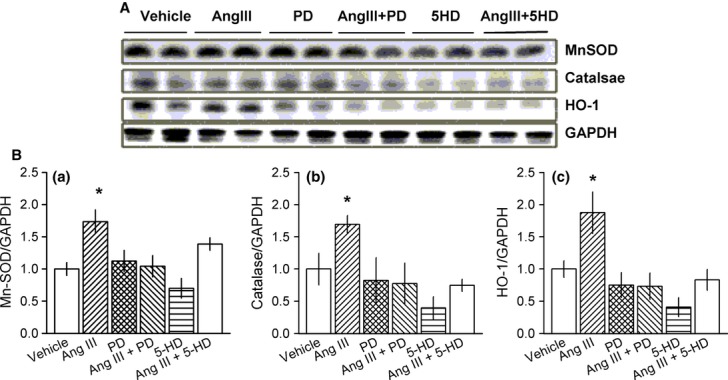
(A) Expressions of Mn-SOD, catalase, and heme oxygenase-1 (HO-1) proteins in the postischemic hearts. The expression level of Mn-SOD, catalase, and HO-1 proteins were determined by western blotting. (B) Densitometric analysis of each protein as compared to GAPDH. Values are mean ± SEM of four to nine hearts of each group. *P < 0.05 vs. I/R group. Mn-SOD, Mn-superoxide dismutase; I/R, ischemia/reperfusion.

**Figure 7:**
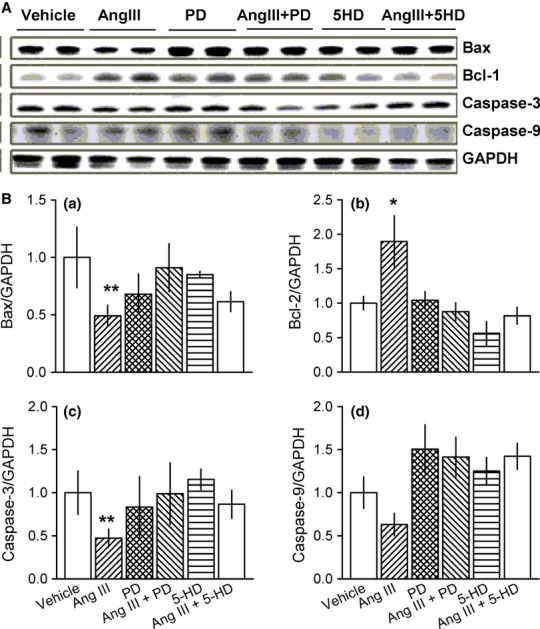
(A) Expressions of Bax, Bcl-2, active caspase-3, and caspase-9 protein levels in postischemic hearts. The expression levels of Bax, Bcl-2, active caspase-3, and caspase-9 proteins were determined by western blotting. (B) Densitometric analysis of each protein as compared to GAPDH. Values are mean ± SEM of 5–11 hearts of each group. *P < 0.05 vs. I/R group. I/R, ischemia/reperfusion.

Figure 6 shows Mn-SOD, catalase, and HO-1 protein levels in ventricular tissues after reperfusion with and without Ang III and PD123319 or 5-HD. Ang III increased Mn-SOD, catalase, and HO-1 protein levels, which were attenuated by pretreatment of PD123319 or 5-HD. Ang III decreased Bax, caspase-3, and caspase-9 protein levels, and increased Bcl-2 protein levels, which were attenuated by pretreatment of PD123319 or 5-HD (Fig. 7). PD123319 and 5-HD itself did not show any significant effects on these protein levels.

## Discussion

In this study, we found that Ang III significantly improved LVDP, LVEDP, and ±dP/dt induced by I/R. Ang III also reduced I/R-induced increases in LDH levels and infarct size. Pretreatment with an inhibitor of AT_2_R or K_ATP_ channel attenuated Ang III-induced cardioprotective effects. Ang III attenuated I/R-induced decreases in Mn-SOD, catalase, and HO-1 levels. In addition, Ang III attenuated increases in Bax, caspase-3, and caspase-9 levels, and a decrease in Bcl-2 level by I/R. Pretreatment with AT_2_R or K_ATP_ channel blocker attenuated the effects of Ang III on antioxidant and apoptotic proteins. These results suggest that the cardioprotective effects of Ang III against I/R injury may be partly due to activating antioxidant enzymes and inhibiting antiapoptotic enzymes through AT_2_R and mitochondrial K_ATP_ channels.

Ang III is generated from Ang II by aminopeptidase A (Carey and Siragy 2003; Reaux et al. 2001) and is known to have similar functions to Ang II in the regulation of aldosterone secretion and blood pressure, even though their potentials are different. In contrast, Ang III appears to have an effect opposite to those of Ang II on sodium excretion (Padia et al. 2009) and ANP secretion via AT_2_R (Park et al. 2013). Based on our results, we propose that Ang III may have possible cardioprotective effects through ANP. In the present experiment, brief exposure to Ang III before ischemia significantly improved ventricular hemodynamics such as LVDP, LVEDP, and ±dP/dt induced by I/R. Ang III also reduced I/R-induced increases in LDH level and infarct size. Pretreatment with AT_2_R antagonist attenuated Ang III-induced cardioprotective effects. It is likely that Ang II and Ang III display similar affinities for both AT_1_R and AT_2_R (Carey and Siragy 2003; Fyhrquist and Saijonmaa 2008). It has been reported that Ang III appears to be a preferred agonist of AT_2_R in the coronary circulation (van Esch et al. 2008; Padia et al.^2009^), kidney (Padia et al.^2009^), and heart (Oh et al. 2011b; Park et al. 2013). Therefore, pretreatment with PD123319 significantly blocked the Ang III-induced cardioprotective effect, suggesting that the effect of Ang III on I/R injury was mediated by AT_2_R.

Next, we explored possible mechanisms of cardioprotection by Ang III. It has been reported that Ang II acts at G(q/11)-coupled receptors to suppress K_ATP_ channel activity in rat arterial smooth muscle (Hayabuchi et al. 2001). However, it is not known whether Ang III suppress or open K_ATP_ channel activity. It has been also reported that the pathology of postischemic cardiac injury is mediated primarily through mitochondrial Ca^2+^ overload and overproduction of reactive oxygen species (ROS) (Sadek et al. 2002), which trigger opening of the mitochondrial permeability transition pore at reperfusion followed by cell death (Kim et al. 2006). In addition, diazoxide, an agonist of the mitochondrial K_ATP_ channel, mimics ischemic preconditioning (Garlid et al. 1997), whereas 5-HD, its antagonist, prevents diazoxide-mediated cardioprotection (Auchampach et al. 1992). The mitochondrial K_ATP_ channel appears to interact with mitochondrial ROS generation (Kim et al. 2005). Therefore, we used 5-HD to modify the cardioprotective effects of Ang III. Pretreatment with 5-HD for 15 min abolished the improvements of LVDP, LVEDP, and ±dP/dt induced by Ang III. Decreased myocardial infarct size and LDH activity induced by Ang III were also attenuated by 5-HD treatment. 5-HD alone had no significant effects on postischemic myocardial dysfunction (Piao et al. 2010). These results suggest that the cardioprotective effects of Ang III against postischemia may be partly mediated through the opening of mitochondrial K_ATP_ channels. Our findings agree with the results of a previous report (Hayabuchi et al. 2001).

The AT_2_R is another counter-regulatory component of the RAS (Carey and Siragy 2003), and stimulation of the AT_2_R by Ang III includes phosphoinositol 3-kinase, Akt, nitric oxide (NO), cGMP, and protein kinase G (Hashikawa-Hobara et al. 2012; Park et al. 2013). In addition, postischemic cardiac dysfunction is associated with decreases in antioxidant enzyme activities as well as increases in oxidative stress (Wattanapitayakul and Bauer 2001; Kim et al. 2005; Tunc et al. 2009) (Piao et al. 2010). In this study, we did not measure ROS formation during reperfusion. However, we expect Ang III may reduce ROS formation during reperfusion. As the interaction of Bcl-2 and Bax plays an important role in regulation of apoptosis, the relationships between antiapoptotic effects conferred by Ang III and the altered expression of Bcl-2 family proteins were investigated. Caspase family proteins are important for cell death. When the cell death program is initiated, pro-caspases, including caspase-3 or caspase-9 are cleaved into active forms. In this study, we demonstrated that Ang III improved I/R-induced decreases in antioxidant enzyme levels such as Mn-SOD, catalase, and HO-1 in isolated rat hearts. Ang III also inhibited I/R-induced increases in Bax, caspase-3, and caspase-9 levels and a decrease in Bcl-2. The activations of antioxidant enzyme levels by Ang III were attenuated by AT_2_R blocker and K_ATP_ channel blocker. These results suggest that the activation of antioxidant enzymes by Ang III may be related to the inhibition of I/R-induced apoptosis through AT_2_R. These results are comparable with those of a previous report showing cardioprotective effects against oxidative stress-induced cell death by K_ATP_ channel opener through antioxidant mechanisms (Kim et al. 2005). There is a possibility that Ang III is a novel function involving K_ATP_ channels. However, the exact relationship between mitochondrial K_ATP_ channel and antioxidant enzymes remains to be elucidated.

It has been reported that dilatation of the coronary arteries is mediated through NO production (Kitakaze et al. 1996), which is impaired by hydroxyl radicals (Paolocci et al. 2001). In this study, a decreased coronary flow by I/R was improved by treatment with Ang III and this effect was mediated via AT_2_R and K_ATP_ channel. Hypoxia (Lawrence et al. 1990) and myocardial ischemia (Arad et al. 1994) stimulate ANP secretion, which causes potent diuresis, natriuresis, and vasorelaxation. In this study, ischemia did not affect the total amount of ANP but Ang III stimulated ischemia-induced ANP secretion at 5 (*P* = 0.07) and 10 min (*P* < 0.05) after reperfusion. Our data are in good agreement with results previously demonstrated in Langendorff perfused and working hearts (Arad et al. 1994). Most of the ANP in the coronary effluent originates from the atrium, and approximately 11% of ANP is secreted from the ventricle (Arad et al. 1994). Atrial distension is the most important factor to stimulate ANP secretion (Cho et al. 1988). However, in nonworking Langendorff-perfused hearts, the atrium is not properly distended. Therefore, global ischemia did not affect the total amount of ANP. In a working heart model with atrial distension, increases in secretion rate and concentration of ANP are more prominent (Arad et al. 1994). Both an improvement of coronary flow and augmentation of ischemia-induced ANP secretion by Ang III may be partly involved in cardioprotective effects of Ang III against I/R injury. An emerging issue in this area is the sympathetic hyperactivity resulting from Ang III release in the brain. While Ang III may be beneficial for cardiac performance, this benefit may be tempered by the impact on neurohormonal modulation of cardiac performance. However, the results showing the potency of Ang III to increase blood pressure is 10 times less than Ang II suggest the impact of sympathetic hyperactivity in the brain is relatively small.

Therefore, we suggest that Ang III may bind to AT_2_R and generate NO and cGMP, which may participate cardioprotection against ischemia in early phase. In addition, Ang III augmented hypoxia-induced ANP secretion and improved a coronary flow, which also participate cardioprotection against ischemia in early phase. Alternatively, NO and cGMP may influence gene transcription and translation of antioxidant and apoptotic proteins, which may mediate protective effects against ischemia in late phase. We conclude that the cardioprotective effects of Ang III against I/R injury may partly derive from the activation of antioxidant enzymes and inhibition of apoptotic enzymes via the AT_2_R and K_ATP_ channels.
